# Interaction of ATP with a Small Heat Shock Protein from *Mycobacterium leprae*: Effect on Its Structure and Function

**DOI:** 10.1371/journal.pntd.0003661

**Published:** 2015-03-26

**Authors:** Sandip Kumar Nandi, Ayon Chakraborty, Alok Kumar Panda, Sougata Sinha Ray, Rajiv Kumar Kar, Anirban Bhunia, Ashis Biswas

**Affiliations:** 1 School of Basic Sciences, Indian Institute of Technology Bhubaneswar, Bhubaneswar, India; 2 Wipro GE Healthcare, Kolkata, India; 3 Department of Biophysics, Bose Institute, Kolkata, India; Kwame Nkrumah University of Science and Technology (KNUST) School of Medical Sciences, GHANA

## Abstract

Adenosine-5’-triphosphate (ATP) is an important phosphate metabolite abundantly found in *Mycobacterium leprae* bacilli. This pathogen does not derive ATP from its host but has its own mechanism for the generation of ATP. Interestingly, this molecule as well as several antigenic proteins act as bio-markers for the detection of leprosy. One such bio-marker is the 18 kDa antigen. This 18 kDa antigen is a small heat shock protein (HSP18) whose molecular chaperone function is believed to help in the growth and survival of the pathogen. But, no evidences of interaction of ATP with HSP18 and its effect on the structure and chaperone function of HSP18 are available in the literature. Here, we report for the first time evidences of “HSP18-ATP” interaction and its consequences on the structure and chaperone function of HSP18. TNP-ATP binding experiment and surface plasmon resonance measurement showed that HSP18 interacts with ATP with a sub-micromolar binding affinity. Comparative sequence alignment between *M*. *leprae* HSP18 and αB-crystallin identified the sequence 49KADSLDIDIE58 of HSP18 as the Walker-B ATP binding motif. Molecular docking studies revealed that β4-β8 groove/strands as an ATP interactive region in *M*. *leprae* HSP18. ATP perturbs the tertiary structure of HSP18 mildly and makes it less susceptible towards tryptic cleavage. ATP triggers exposure of additional hydrophobic patches at the surface of HSP18 and induces more stability against chemical and thermal denaturation. *In vitro* aggregation and thermal inactivation assays clearly revealed that ATP enhances the chaperone function of HSP18. Our studies also revealed that the alteration in the chaperone function of HSP18 is reversible and is independent of ATP hydrolysis. As the availability and binding of ATP to HSP18 regulates its chaperone function, this functional inflection may play an important role in the survival of *M*. *leprae* in hosts.

## Author Summary

*Mycobacterium leprae* is the causative organism of the disease so called leprosy. It posses global health challenge due to the emergence of drug resistant *M*. *leprae* bacilli. In search for new drug targeting strategies, study of bacterial energy metabolism is significant. Reports claim that *M*. *leprae* generates ATP, an important energy metabolite, which is very much required for its own biochemical activities and unlike other organisms, it needn’t require host species as a source of ATP. The growth and survival of these pathogenic bacteria in infected hosts are supported by an immunodominant antigenic membrane protein called HSP18. Hereby we report for the first time evidences of HSP18 and ATP interaction *in vitro* and quantified the same. We also identified the Walker-B ATP binding motif in *M*. *leprae* HSP18. Our study reveals that HSP18 when interacts with ATP, undergoes an enhancement in its anti-aggregation property. We demonstrate here a novel report on ability of HSP18 to prevent thermal inactivation of enzymes which is also enhanced in presence of ATP. Our study provides new insights into the effect of ATP on the structure and chaperone function of *M*. *leprae* HSP18 which may be critical for survival of *M*. *leprae* bacilli. This may provide a new basis for the development of new drug targeting strategy such as ATP competitive antibiotics/inhibitors in context of effective treatment of leprosy.

## Introduction

*Mycobacterium leprae* (*M*. *leprae*) HSP18 is an immunodominant antigen and membrane bound protein [1–2]. It is specifically activated during intracellular growth and is involved in the survival of *M*. *leprae* pathogen within macrophages [3]. Moreover, HSP18 is a class 3 (acr 3) heat shock protein (HSP) [4] and shares high degree of sequence homology with homologs of class 3 small heat shock proteins (sHSPs) found in other mycobacterial species [1]. Similar to all sHSPs, its sequence has been divided into three distinct regions: N-terminal region comprising residues 1–38, a highly conserved central “α-crystallin domain” comprising residues 39–121 and a flexible C-terminal tail with residues 122–148 [5]. Recently, we have shown that *M*. *leprae* HSP18 is a major β-sheet protein and exist as an oligomeric assembly (29-mer) [5]. Along with others, we have also shown that it can exhibit molecular chaperone function [5–6]. Our study demonstrated that HSP18 can prevent the aggregation of different stressed client proteins and refold chemically denatured enzymes to its native state [5]. It is believed that the molecular chaperone function of HSP18 may play a crucial role in the long term survival of *M*. *leprae* pathogen in infected hosts [6].

*M*. *leprae* is an intracellular obligatory parasite that is non-culturable *in vitro* [7]. So, it is difficult to get an idea about its pathophysiology. Therefore, several attempts were made to understand its energy metabolism which would give an idea about its nutritional requirements and its relation with its host species. Lee and his coworkers revealed that *M*. *leprae* generates its own ATP when incubated *in vitro* [8]. They further confirmed that *M*. *leprae* did not derive ATP from the host species as it was unable to uptake exogenous ATP during *in vitro* incubation. Another report by the same group clearly demonstrated the presence of adenyl kinase, an enzyme that catalyses the reaction 2 ADP ⇆ ATP + AMP, in cell free extracts of *M*. *leprae* [9]. This confirms that *M*. *leprae* has its own mechanism to generate ATP which is very much required for its own biochemical activities and unlike other organisms, it does not require host species as a source of ATP.

ATP is an important small molecule, which readily stores chemical energy in two of its phosphate bonds. Its role in altering the molecular chaperone function of heat shock proteins is well documented. Many important classical chaperones of large heat shock protein family function in an ATP dependent way [10–12] where its hydrolysis is required for chaperone mediated refolding of substrate proteins [12–13]. In contrast, the role of ATP on the chaperone function of sHSPs is controversial. Mostly, many sHSPs are usually believed to be ATP independent chaperones [14–15]. But, recently it has been demonstrated that the structure and chaperone function of different sHSPs are modulated by ATP. Quenching of intrinsic tryptophan fluorescence, perturbation in the surface hydrophobicity and tryptic digestion studies have revealed that ATP induces conformational changes in α-crystallin and *Mycobacterium tuberculosis* HSP16.3 [16–19], which eventually enhances their chaperone function [18, 20]. In contrast, experiments with tobacco HSP18 have shown that ATP has an opposite effect. It reduces chaperone activity of this small heat shock protein [21]. Decreased surface hydrophobicity appears to be responsible for such ATP effect.

Recently, autophosphorylation of *M*. *leprae* HSP18 has been shown by Dharmalingam and his co-workers [22]. They proposed that such autophosphorylation may be the basis for the survival of *M*. *leprae* under various stressed conditions. Interestingly, leprosy patients are often investigated for viability of *M*. *leprae* by PCR method where this 18 kDa gene is used as target and ATP assay method [23–27]. As HSP18 is harbored by *M*. *leprae* pathogen; there is a possibility that this small heat shock protein is also exposed to such intracellular ATP environment. But, the information about the interaction between *M*. *leprae* HSP18 and ATP has not been reported in the literature. Moreover, if HSP18 interacts with ATP, its effect on the structure and chaperone function of this mycobacterial sHSP is yet to be explored.

In this study, we have identified the location of ATP binding in HSP18 using molecular docking studies. Subsequently, we quantified the interaction between *M*. *leprae* HSP18 and ATP using surface plasmon resonance (SPR) and TNP-ATP binding experiments. We have also investigated the effect of ATP on the structure and chaperone function of *M*. *leprae* HSP18. We report here that ATP improves the chaperone function of HSP18. Besides, we account here the molecular basis behind the enhanced chaperone activity of *M*. *leprae* HSP18 in the presence of ATP using several biochemical and biophysical studies.

## Materials and Methods

### Materials

Dithiothreitol (DTT), 4,4'-dianilino-1,1'-binaphthyl-5,5'-disulfonic acid dipotassium salt (bis-ANS), urea, isopropyl β-D-1-thiogalactopyranoside (IPTG), adenosine triphosphate (ATP), adenosine diphosphate (ADP), adenosine monophosphate (AMP), adenosine 5′-[γ-thio] triphosphate tetralithium salt (ATP-γS), 2′,3′-O-(2,4,6-Trinitrophenyl) adenosine 5′-triphosphate tetrasodium salt (TNP-ATP), lysozyme, citrate synthase (CS), were obtained from Sigma Chemical Co. (St. Louis, MO, USA). Malate dehydrogenase (MDH), oxaloacetic acid (OAA), nicotinamide adenine dinucleotide (reduced) disodium salt (NADH), trypsin, buffer salts (phosphate, tris, etc.) were from Sisco Research Laboratories, India. All other chemicals were of analytical grade.

### Expression and purification of recombinant *M*. *leprae* HSP18

Plasmid was obtained as a gift from Prof. Dharmalingham (Madurai Kamraj University, Madurai, India). Methods of expression and purification of wild-type *M*. *leprae* HSP18 have been described previously [5]. Concentration of HSP18 was determined spectrophotometrically by measuring absorbance at 278 nm using extinction coefficient of 0.4 (mg/ml)^-1^cm^-1^. Concentration of this protein was also determined using Bradford assay. All the biophysical assays were performed with at least three independent protein preparations.

### Binding of TNP-ATP to HSP18

HSP18 (2.5 μM) was incubated with equimolar concentration of TNP-ATP in 50 mM phosphate buffer (pH 7.5) for 1 hr. at 25°C. Fluorescence emission spectra of this incubation mixture were recorded between 500–600 nm at 25°C using a spectrofluorometer from Horiba Jobin Mayer, USA (Fluoromax 4P). To the above assay mixture, 0.25 mM ATP was added and fluorescence intensity of TNP-ATP bound HSP18 complex was also measured. The excitation wavelength used was 403 nm. The excitation and emission band-passes were 5 nm each. Data were collected at 0.5 nm wavelength resolution.

In an another experiment, HSP18 (2.5 μM) in 50 mM phosphate buffer (pH 7.5) was titrated with 300 μM TNP-ATP. This fluorescence intensity of bound TNP-ATP was recorded at 538 nm with an excitation wavelength 403 nm using the same spectrofluorometer mentioned above. The temperature of the samples was maintained at 25°C by using a circulating water bath attached to the spectrofluorimeter. A reverse titration was also performed with 0.1 μM of TNP-ATP titrated against 2.5 mg/ml HSP18. This reverse titration was used to convert the fluorescence intensity change into bound TNP-ATP, similar to an approach taken by Cardamone and Puri for reverse titration of bis-ANS with porcine somatotrophin [28]. To calculate the number of TNP-ATP molecules bound per molecule of monomeric HSP18 (n) and the dissociation constant (K_d_) for the interaction between TNP-ATP and HSP18, these forward and reverse titration data were analysed by the Scatchard equation:

$$\left[Bound\right]=n-K_{d}\frac{\left[Bound\right]}{\left[Free\right]}$$ (1)

where [Bound] is the number of moles of substrate bound per mole of chaperone, [Free] is the concentration of free substrate.

### Surface plasmon resonance (SPR) measurements

Surface plasmon resonance of carboxymethyl-dextran coupled HSP18 was measured using a Biacore T200 instrument (GE Healthcare). Briefly, carboxymethyl-dextran (CM5) sensor chips were activated using equimolar amounts of N-hydroxysuccinimide and N-ethyl-N’-(3-dimethylaminopropyl)-carbodiimide hydrochloride for 7 min (Amine Coupling Kit; Biacore). *M*. *leprae* HSP18 (0.075 mg/ml) was coupled to the chip in a buffer containing 10 mM acetate (pH 4.5) at a flow rate of 10 μl/min. To deactivate any unreacted esters and to remove any remaining unbound protein, the surface of the flow cell was then blocked with ethanolamine for 7 min. A second flow cell was activated and deactivated in the same way to yield a blank surface. All analyte solutions (adenine nucleotides—AMP, ADP, ATP and ATP-γS) were prepared in degassed, filter-sterilized PBS buffer with or without 3.5 mM MgCl_2_. The same solution was used as running buffer during binding experiments. For the binding of adenine nucleotides with immobilized HSP18, the concentration of adenine nucleotides was varied between 0.1 μM and 100 μM. The binding was carried out at 25°C with a flow rate of 30 ml/min, and data were collected for 180 s of association and 180 s of dissociation. After the completion of the dissociation step, surface regeneration was accomplished with a 1 mini injection of 10 mM glycine HCl solution pH 2.5 at a flow rate of 50 ml/min. To analyze the data, the assay flow cell was subtracted by the control (blank) flow cell to eliminate non-specific interaction. K_d_ was calculated by BIACORE T200 evaluation software with nonlinear fitting, the 1:1 (Langmuir) binding model.

### Chaperone activity assays

The chaperone activity of HSP18 in the presence or absence of ATP and its non-hydrolysable analog (ATP-γS) was determined with two client proteins: lysozyme and citrate synthase (CS). All assays were performed with the aid of Perkin Elmer Lambda 35 UV spectrophotometer.

#### Chemically induced aggregation assay

HSP18 was preincubated with 0.01–3 mM ATP or 1 mM ATP-γS for 1 hr at 25°C. Then, aggregation of lysozyme (0.05 mg/ml), in absence and presence of 0.05 mg/ml HSP18 preincubated with/without ATP/ATP-γS, was initiated by addition of 20 mM DTT at 37°C in 50 mM phosphate buffer pH 7.5. Light scattering at 400 nm was monitored for 1 hr in the kinetic mode at 37°C.

#### Thermally induced aggregation assay

HSP18 was preincubated with 0.01–1 mM ATP or 1 mM ATP-γS for 1 hr at 25°C. Native CS (0.06 mg/ml) was heated at 43°C in 50 mM phosphate buffer (pH 7.5) in the absence or presence of preincubated 0.06 mg/ml HSP18 with/without ATP/ATP-γS. The aggregation of CS was monitored at 400 nm for 1 hr in the kinetic mode at 43°C.

### Thermal deactivation assay

Thermal deactivation of 10 nM MDH in 50 mM phosphate buffer, pH 7.5 was initiated by heating the enzyme at 43°C in the absence or presence of 30 μM HSP18 preincubated with/without 0.01–3 mM ATP or 1 mM ATP-γS. Enzyme activity of MDH was measured by taking aliquots from the assay mixture which was incubated at 43°C for 10 mins. Enzyme activity of MDH was assayed using nicotinamide adenine dinucleotide reduced (NADH) and oxaloacetic acid (OAA) as described previously [29]. To check whether the effect is specific to HSP18, we performed the same experiment in presence of 30 μM BSA.

### Circular dichorism (CD) measurements

Far-UV CD spectra of different HSP18 solutions were measured at 25°C using a spectropolarimeter (Chirascan, Applied Photophysics, Leatherhead, UK) equipped with a peltier system. Before recording the spectra, HSP18 was incubated without/with 1 mM ATP at 25°C for 1 hr. Spectra were collected from 195–260 nm using a quartz cell with 1 mm path length. Proteins (0.2 mg/ml) were dissolved in 10 mM phosphate buffer, pH 7.5.

The near-UV CD spectra were also measured at 25°C using the same spectropolarimeter after incubating HSP18 (0.5 mg/ml in 50 mM phosphate buffer, pH 7.5) without/with 1 mM ATP at 25°C for 1 hr. The reported spectra in both far- and near-UV CD experiment were the average of 5 scans.

### Intrinsic tryptophan fluorescence measurements

HSP18 (0.05 mg/ml) in 50 mM phosphate buffer, pH 7.5, was incubated at 25°C for 1 hr in the absence and presence of 1 mM ATP. The intrinsic tryptophan fluorescence spectra of proteins (0.05 mg/ml) in 50 mM phosphate buffer, pH 7.5 at 25°C were recorded using a spectrofluorometer from Horiba Jobin Mayer, USA (Fluoromax 4P). The excitation wavelength was set to 295 nm and the emission spectra were recorded between 310–400 nm. Data were collected at 0.5 nm wavelength resolution.

### Hydrodynamic radius (R_H_) measurements by DLS

*M*. *leprae* HSP18 (0.5 mg/ml, in 50 mM phosphate buffer, pH 7.5) preincubated without/with 1 mM ATP was filtered through 0.22 μm disk membrane. Experiment was performed at 25°C. Hydrodynamic radius of HSP18 oligomers was measured by dynamic light scattering (DLS) employing a Zetasizer Nano S (Malvern Instruments). A 4-mW He—Ne laser (633 nm) with a fixed detector angle of θ = 173° was used. The time-dependent autocorrelation function was acquired every 60s, with twelve acquisitions for each run. Each data is an average of four such acquisitions. R_H_ was calculated of using the Stokes—Einstein equation:

$$R_{H}=\frac{k_{B}T}{6\pi \eta D}$$ (2)

Where, k_B_ is Boltzmann’s coefficient, T is absolute temperature, η is the viscosity of the medium and D is translational diffusion coefficient of the particles [30].

### Determination of surface hydrophobicity

The surface hydrophobicity of the HSP18 in the presence and absence of ATP and ATP-γS was measured with bis-ANS, a specific hydrophobic probe [18]. HSP18 (0.05 mg/ml) in 50 mM phosphate buffer, pH 7.5, was incubated for 1 hr at 25°C in the presence and absence of 1 mM ATP and ATP-γS. bis-ANS (10μM) was added to HSP18 and the mixture was incubated for 60 mins at 25°C. Fluorescence emission spectra were recorded between 450–550 nm at 25°C using an excitation wavelength of 390 nm. The excitation and emission band-passes were 2.5 and 5 nm, respectively. Data were collected at 0.5 nm wavelength resolution. We also did this experiment in presence of 0.01 mM ATP.

### Trypsin digestion experiment

The effect of ATP on the structural compactness of HSP18 was measured by comparing the trypsin digestibility of HSP18 [5]. HSP18 (0.5 mg/ml in 50 mM phosphate buffer, pH 7.5) in the absence or presence of 1 mM ATP, were incubated with trypsin (at a ratio of 100:1, w/w) at 37°C. Aliquots were withdrawn after different periods of digestion and the reaction was stopped immediately by adding soybean trypsin inhibitor. SDS-PAGE of the digested proteins was performed under reducing conditions in a Bio-Rad Mini- PROTEAN 3 electrophoresis setup using a linear 8 to 16% gradient polyacrylamide gel. Gels were photographed with gel documentation system (UVP Gel Doc It^2^, UK) and the densitometry analysis was performed with the help of Vision LS software provided with the gel documentation system.

### Estimation of structural stability

#### Chemical denaturation experiment

The structural stability of HSP18 was also determined by equilibrium chemical denaturation experiment. HSP18 (0.05 mg/ml in 50 mM phosphate buffer, pH 7.5) in the presence or absence of 1 mM ATP was incubated separately with various urea concentrations (0–7 M) for 18 hr at 25°C. Intrinsic tryptophan fluorescence spectra of all samples were recorded between 310–400 nm region using an excitation wavelength of 295 nm. The equilibrium unfolding profile was fitted according to the three state model as described earlier [5, 18].

#### Thermal denaturation experiment

The structural stability of HSP18 in presence or absence of 0.01/1 mM ATP was also determined using thermal induced unfolding experiments in spectropolarimeter (Chirascan, Applied Photophysics, Leatherhead, UK) equipped with a peltier system. The change in ellipticity at 222 nm was recorded between 25 to 85°C in a quartz cell with a path length of 1 mm, allowing the samples to equilibrate at each temperature. Heating rate was set to 0.5°C/min. Data were recorded at an interval of 2°C. The protein concentration was 0.2 mg/ml in 50 mM phosphate buffer (pH 7.5). Mid-point transition or T_m_ was calculated using sigmoidal analysis as described previously [5].

### Theoretical studies for identifying ATP bind motif/sites in HSP18

A homology model was prepared for HSP18 (Acc. No. ML1795) using the protein primary sequence which was obtained from the SWISSPROT database [31]. Prime *v3*.*4* module [32] of Schrӧdinger molecular modeling suite was used for the model building. The three-dimensional coordinates of wheat HSP16.9 (PDB code: 1GME_A) was selected as template for the modeling building, which share 47% sequence similarity with HSP18. The fold recognition method was used in the module, thereby the folding of protein backbone atoms were copied for further refinement. The side chain optimization was performed and minimization of the residues was performed for those, which has not been derived from the template. The entire procedure was performed using OPLS_2005 all-atom force field [33]. Consideration of the solvation effect and energies were performed using Surface Generalized Born (SGB) continuum solvation model. The final acceptance of the model was done with the help of energy minimization using Polak-Ribiere conjugate gradient (PRGC) method, where maximum iteration steps of 2500 was used. The convergence threshold used was 0.05. The final validation of the model was performed using Ramachandran plot as similar to previous literature [34].

ATP molecule was sketched and prepared using Ligprep module *v*. *2*.*8*, where bond order, bond length, atomic charge, target pH and other similar parameters were used as similar to default settings. Docking of ATP into the three dimensional structure of HSP18 (obtained through the homology modeling using coordinates of wheat HSP16.9, PDB code: 1GME_A) was performed using Glide module *v*. *6*.*1* [35]. Standard Precision (SP mode) of docking calculation (available in Glide module *v*. *6*.*1*) was used for obtaining the results. Other calculation such as solvent assessable surface area (SASA) was computed using NACESS standalone tool *v2*.*1*.*1* (http://www.bioinf.manchester.ac.uk/naccess/). Prime *v3*.*4* module was also used to perform the multiple sequence alignment of Walker-B ATP binding motif of human αB-crystallin and HSP18. The sequence alignment between different protein pairs (HSP18 and αB-crystallin or HSP18 and HSP16.3) was also performed using ClustalW programme.

### Statistical analysis

All the values are represented as means ± the standard deviation (n = 3). We used Student’s *t*-test to analyze the statistical differences between groups. *p-values* of ≤ 0.05 were considered statistically significant.

## Results and Discussion

Large HSPs interact differently with adenine nucleotides (specifically towards ATP). DnaK and Cpn60 bind ATP with the dissociation constant (K_d_) values of 0.22 and 10 μM, respectively [36–37]. Several other large HSPs including HSP70, HSP104 and ClpB, which exhibit chaperone function in an ATP dependent way, bind ATP/other adenine nucleotides with dissociation constant (K_d_) values in sub-micromolar range [38–39]. Though ATP influences the chaperone function of several sHSPs, their interaction with ATP is scarcely characterized/quantified in the literature. Palmisano *et al*. reported that α-crystallin interacts with ATP with a dissociation constant (K_d_) value of ~128 μM (K_a_ = 7.84 x 10^3^ M^-1^) [16]. Since, very limited knowledge exists in the literature regarding the interaction between *M*. *leprae* HSP18 and intracellular ATP which is generated by the pathogen itself, we took an attempt to measure the affinity of ATP towards *M*. *leprae* HSP18.

### TNP-ATP, a fluorescent ATP analog, binds to *M*. *leprae* HSP18

**Fig 1:**
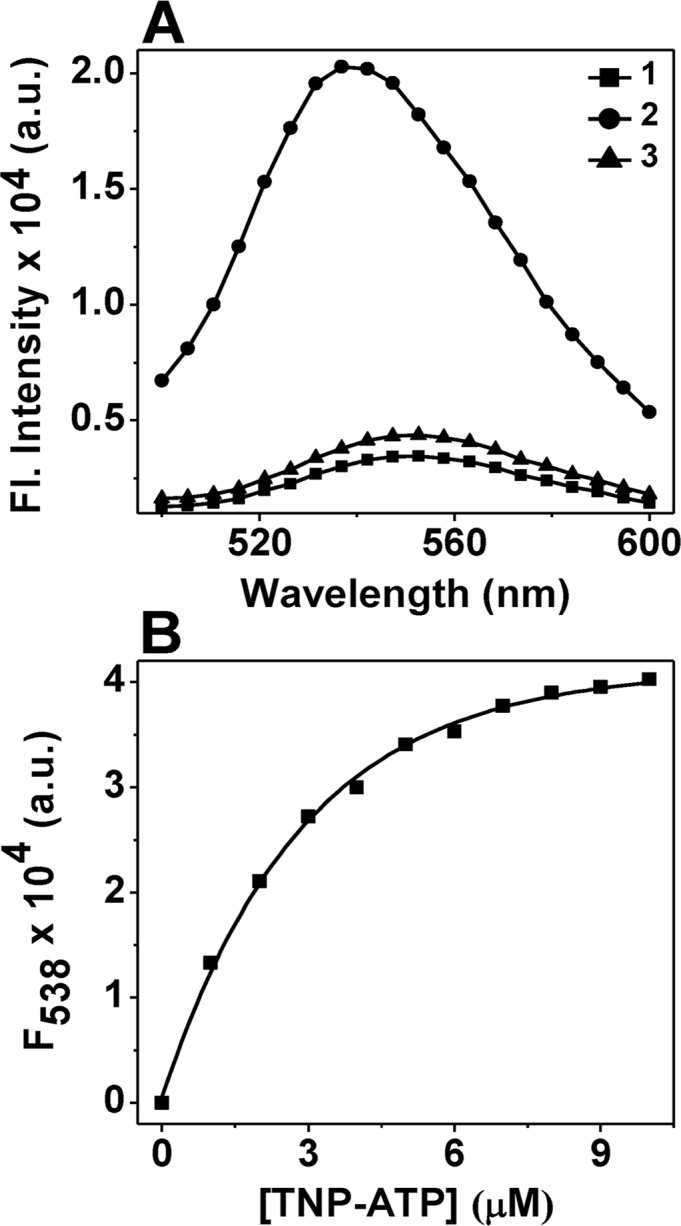
Fluorescence spectra of TNP-ATP bound to *M*. *leprae* HSP18. **(A)** Fluorescence emission spectra of TNP-ATP alone (trace 1); TNP-ATP+HSP18 (trace 2); Addition of 0.25 mM ATP to the HSP18-TNP-ATP complex (trace 3). Protein concentration used was 2.5 μM and TNP-ATP concentration was 2.5 μM. Excitation wavelength used was 403 nm and fluorescence emission spectra were recorded from 500–600 nm. **(B)** Fluorescence titration of 2.5 μM *M*. *leprae* HSP18 was carried with varying concentration of TNP-ATP with fluorescence monitored at λ_exc_ = 403 nm and λ_em_ = 538 nm.

In this study, we used TNP-ATP, a fluorescent ATP analog, for investigating the binding affinity/interaction of ATP with *M*. *leprae* HSP18. This probe is commonly used for examining the nucleotide binding of various proteins [40–41]. Structural characterization of the probe bound to different bacterial histidine kinases has revealed that TNP-ATP occupies their nucleotide-binding pocket/domain [40–41]. Free TNP-ATP exhibits very weak fluorescence in buffer solutions. But when it binds to proteins, its fluorescence intensity increases several folds [42]. We observed that the fluorescence intensity of 2.5μM TNP-ATP increases ~6 fold in the presence of equimolar concentration of *M*. *leprae* HSP18, with a λ_max_ of 538 nm (Fig. 1A, trace 2). ATP can efficiently displace TNP-ATP from the nucleotide binding pocket in protein: TNP-ATP complexes, resulting in decrease of TNP-ATP fluorescence which was also observed in case of HSP18. When 0.25 mM ATP was added to the HSP18-TNP-ATP complex at 25°C, significant reduction in TNP-ATP fluorescence intensity was observed (Fig. 1A, trace 3). We also titrated HSP18 (2.5 μM) with increasing concentration of TNP-ATP (0–10μM) (Fig. 1B). The titration curve indicated that the fluorescence intensity of TNP-ATP bound to protein increased gradually upon the addition of TNP-ATP in the solution and the titration curve reached saturation upon the addition of ~8 μM TNP-ATP. In order to determine the dissociation constant (K_d_) and the stoichiometry of binding (n) of TNP-ATP per mol of HSP18 subunit, the binding data were analyzed by the Scatchard equation (Equation 1). Prior to this analysis, a reverse titration of TNP-ATP by HSP18 was performed to convert the fluorescence intensity change into bound TNP-ATP. The analysis revealed that 19 TNP-ATP molecules are interacting with 29-mer *M*. *leprae* HSP18 with a dissociation constant (K_d_) value of 0.73 μM.

### Estimation of binding affinity of ATP and other nucleotides (AMP, ADP and ATP-γS) using SPR technique

**Fig 2:**
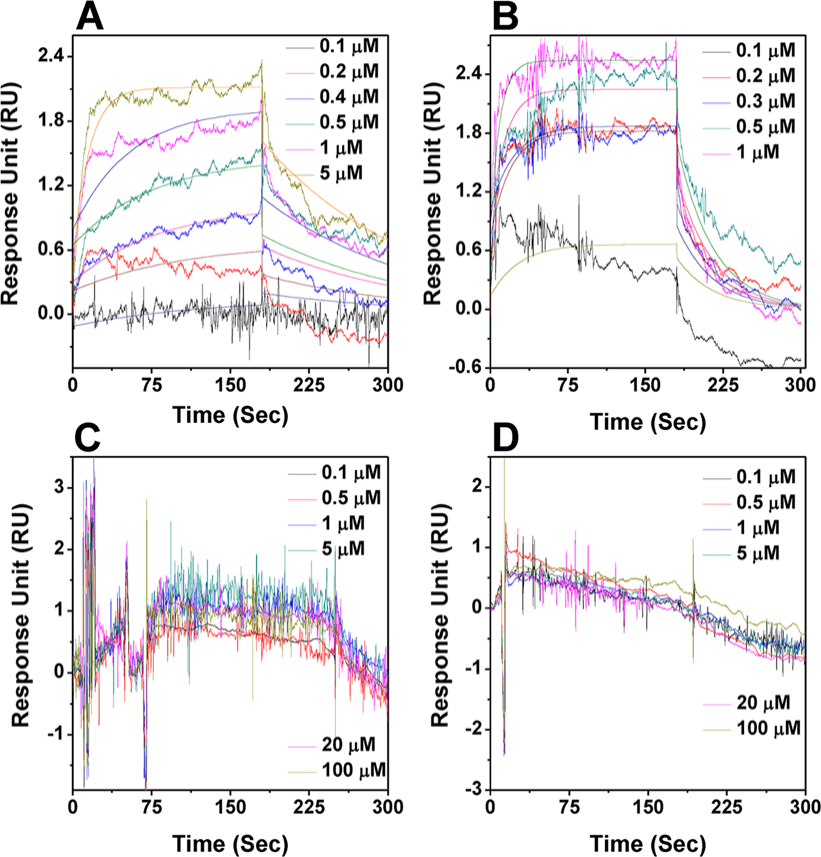
SPR analysis of the interaction of adenine nucleotides to immobilized. *M*.*leprae* HSP18 interactions. Sensorgrams (solid line) of the binding of **(A)** ATP (0.1–5 μM); **(B)** ATP-γS (0.1–1 μM); **(C)** ADP (0.1–100 μM) and **(D)** AMP (0.1–100 μM) to *M*. *leprae* HSP18 covalently captured on a sensor chip at 25°C.

To get the idea about the binding affinity of ATP and other adenine nucleotides (AMP, ADP and ATP-γS) precisely, we performed the surface plasmon resonance (SPR) experiments. This method so employed is used to measure changes in refractive index when adenine nucleotides passes over the HSP18 immobilized on the surface of carboxymethylated dextran. Fig. 2 shows changes in HSP18 surface plasmon resonance on interaction with various adenine nucleotides. At 25°C, the interaction between ATP and immobilised HSP18 was noticeable (Fig. 2A) and the binding affinity of this interaction was found with a dissociation constant (K_d_) value of 0.59 μM. HSP18 also interacted with the non-hydrolyzable analog of ATP (ATP-γS) with almost similar K_d_ value (0.33 μM) (Fig. 2B). The magnitude of binding affinity remained unaltered even when the adenine nucleotide solutions (ATP) were devoid of MgCl_2_ (S1A Fig.). However, no interaction was detected between ADP or AMP with HSP18 even at 100 μM (Fig. 2C and D). Thus, it is evident from the above results that amongst the four adenine nucleotides, ATP and its non hydrolysable analog interacts with HSP18. Therefore, we studied only the effect of "HSP18-ATP/ATP-γS interaction" on the structure and chaperone function of *M*. *leprae* HSP18.

### ATP binding improves the aggregation and thermal deactivation prevention ability of HSP18

**Fig 3:**
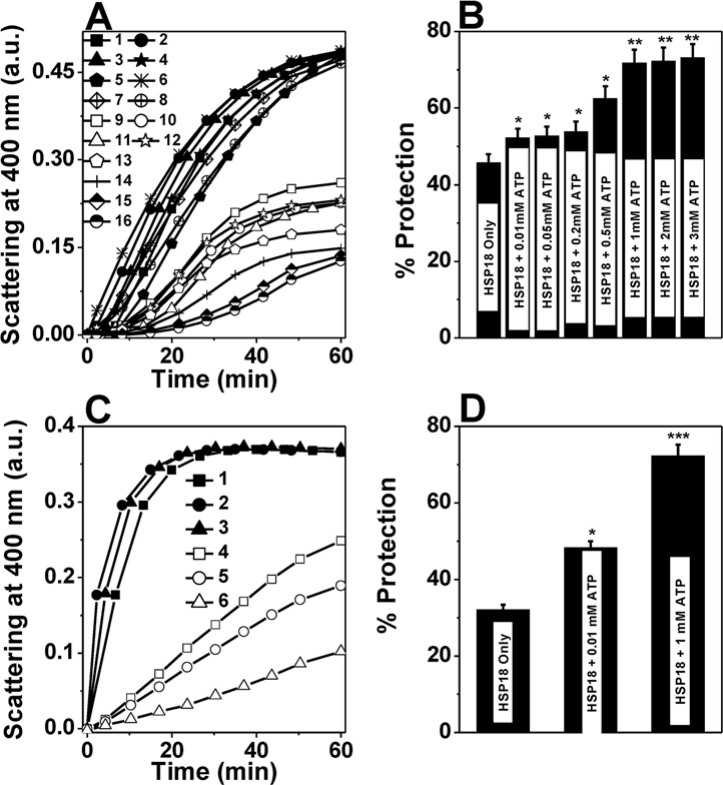
Effect of ATP on the chaperone activity of *M*. *leprae* HSP18. **(A)** DTT-induced aggregation of 0.05 mg/ml lysozyme (Lys) in the absence and presence 0.05 mg/ml HSP18 preincubated with 0.01–3 mM ATP at 25°C. Aggregation was initiated by adding 20 mM DTT and scattering at 400 nm was monitored at 37°C. Trace 1: Lys alone; Trace 2: Lys + 0.01 mM ATP; Trace 3: Lys + 0.05 mM ATP; Trace 4: Lys + 0.2 mM ATP;Trace 5: Lys + 0.5 mM ATP;Trace 6: Lys + 1 mM ATP; Trace 7: Lys + 2 mM ATP;Trace 8: Lys + 3 mM ATP; Trace 9:Lys + HSP18; Trace 10: Lys + HSP18 preincubated with 0.01 mM ATP; Trace 11: Lys + HSP18 preincubated with 0.05 mM ATP; Trace 12: Lys + HSP18 preincubated with 0.2 mM ATP; Trace 13: Lys + HSP18 preincubated with 0.5 mM ATP; Trace 14: Lys + HSP18 preincubated with 1 mM ATP; Trace 15: Lys + HSP18 preincubated with 2 mM ATP; and Trace 16: Lys + HSP18 preincubated with 3 mM ATP; **(B)** Percent protection ability of *M*. *leprae* HSP18 against lysozyme aggregation in the absence and presence of 0.01–3.0 mM ATP at 37°C. Data are means ± the standard deviation from triplicate determinations; **(C)** Thermal aggregation of CS (0.06 mg/ml) at 43°C. Prior to this measurement, HSP18 was preincubated with or without 0.01/1 mM ATP for 1 hr at 25°C. Trace 1: CS alone; Trace 2: CS + 0.01 mM ATP; Trace 3: CS + 1 mM ATP; Trace 4: CS + HSP18; Trace 5: CS + HSP18 preincuabted with 0.01 mM ATP; Trace 6: CS + HSP18 preincuabted with 1 mM ATP; **(D)** Percent protection ability of *M*. *leprae* HSP18 against CS aggregation in the absence and presence of 0.01/1 mM ATP at 43°C. Data are means ± the standard deviation from triplicate determinations.*p< 0.05, **p< 0.005 and ***p< 0.0005.

Firstly, the chaperone activity of recombinant HSP18 was studied in the absence or presence of ATP (0.01–3 mM) using DTT induced aggregation of lysozyme at 37°C. The effects of different concentrations of ATP on the aggregation of lysozyme in the presence or absence of HSP18 are shown in Fig. 3A. At 1:1 (w/w) ratio of HSP18 to lysozyme, 45% protection against aggregation was observed in the absence of ATP (Fig. 3A, trace 9 and 3B). In the presence of 0.01–0.2 mM ATP, protection was increased to ~52–54% (Fig. 3A, traces 10–12 and 3B). When ATP concentration was further increased to 1 mM, a further increase in the protection against lysozyme aggregation was observed (~72%) (Fig. 3A, trace 14 and 3B). No further enhancement in the protection ability of HSP18 was observed in presence of 2 and 3 mM ATP (Fig. 3A, traces 15–16 and 3B). Therefore, it is quite evident from here that the interaction between ATP and *M*. *leprae* HSP18 has profound influence on the aggregation prevention ability of HSP18. This interaction increases the chaperone function of this mycobacterial sHSP.

We used another client protein (CS) to reconfirm this fact. We compared the chaperone activity of *M*. *leprae* HSP18 heated with CS at 43°C in the presence and absence of 0.01/1 mM ATP (Fig. 3C). In absence of ATP, HSP18 inhibited protein aggregation efficiently (~30%) at a chaperone:client protein ratio of 1:1 (w/w) (Fig. 3C, trace 4 and 3D). While, at the same ratio, upon the interaction with 1 mM ATP, the protection ability of HSP18 increased to ~70% (Fig. 3C, trace 6 and 3D). Even at very low ATP concentration (0.01 mM), the chaperone function of HSP18 was enhanced (~16%) (Fig. 3C, trace 5 and 3D). We also noticed the enhancement in the aggregation prevention ability of HSP18 in presence of 0.2 and 0.5 mM ATP (S1B Fig.). Thus our findings clearly revealed that the protection ability of *M*. *leprae* HSP18 was enhanced in the presence of ATP.

**Fig 4:**
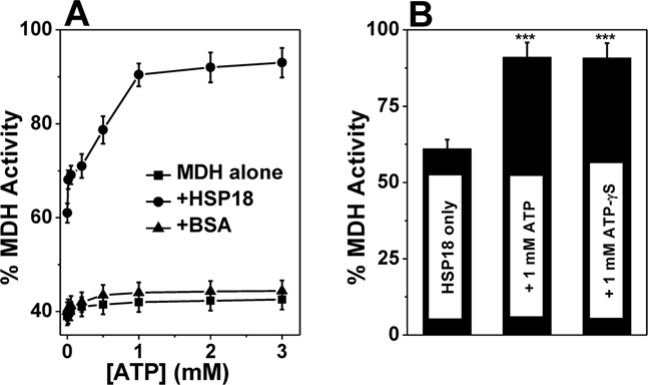
Effect of ATP on the thermal deactivation prevention ability of *M*. *leprae* HSP18. The enzyme activity of MDH was measured in absence and presence of 30 μM HSP18 preincubated with/without **(A)** 0.01–3 mM ATP; **(B)** 1 mM ATP/ATP-γS during its thermal denaturation at 43°C. The assay also performed with 30 μM BSA alone or preincubated with 0.01–3 mM ATP. ***p<0.0005.

Apart from preventing the aggregation of different stressed client proteins, HSPs are also known to prevent the loss of enzymatic activity under thermal stress. This ability of HSPs is also influenced by ATP. Zietara *et al*. reported that DnaK could protect LDH from thermal inactivation and this protection increased by ~10% in presence of 2 mM ATP [43]. Hartman *et al*. revealed that GroEL (along with GroES) failed to prevent MDH from thermal inactivation. It could prevent the thermal inactivation of MDH only when ATP was included in the system [44]. We already demonstrated that *M*. *leprae* HSP18 can prevent aggregation of different stressed substrate proteins and also can reactivate enzyme activity of denatured MDH [5]. But, whether it can prevent enzymes from thermal inactivation, is still not known. In order to explore this functionality of HSP18 and to assess whether ATP altered its ability to prevent thermal deactivation of enzymes, we incubated 10 nM MDH for 10 mins at 43°C and measured its activity. Fig. 4A shows that MDH alone quickly lost its activity and retained only 39% activity. However, in the presence of HSP18 (30 μM), the loss of MDH activity was reduced and it retained 61% of its enzymatic activity. When 0.01–1 mM ATP was added to the assay mixture along with 30 μM HSP18, the loss of MDH activity was further retarded and MDH was able to retain 68% to 90% of its activity. Marginal improvement in the retention of MDH activity was observed upon the addition of HSP18 and higher amount of ATP (2 and 3 mM). To check the specificity, a parallel experiment was executed where HSP18 was replaced with BSA and was incubated in absence and presence of 0.01–3 mM ATP. It was found that, unlike HSP18, BSA failed to prevent the thermal inactivation of MDH under similar experimental conditions (Fig. 4A). Together, our findings indicate that like other HSPs, HSP18 also has the ability to prevent thermal deactivation of enzyme. Also, our data indicated that ATP enhanced this ability of *M*. *leprae* HSP18. Both *in vitro* aggregation and thermal deactivation assays revealed that the enhancement in the chaperone function of HSP18 was saturated in presence of 1 mM ATP. Therefore, we decided to use this ATP concentration (1 mM) in all the subsequent experiments.

### HSP18 does not require ATP hydrolysis for its enhanced chaperone function

**Fig 5:**
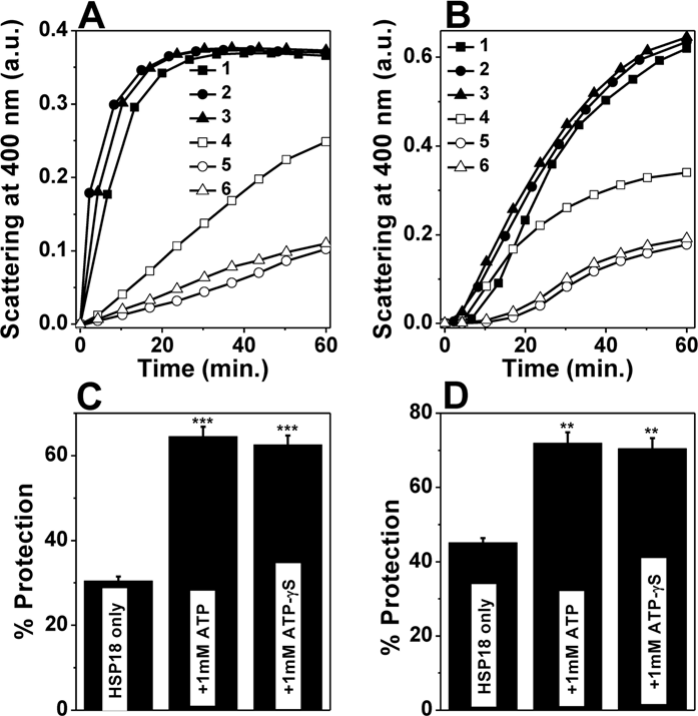
Enhancement of chaperone activity of *M*. *leprae* HSP18 induced by ATP and its non-hydrolysable analog, ATP-γS. Thermal aggregation of 0.06 mg/ml CS at 43°C **(panel A)** and DTT-induced aggregation of 0.05 mg/ml lysozyme at 37°C **(panel B)** in the absence or presence of different HSP18 preparations. Trace 1:Client protein (CP) alone; Trace 2: CP + 1 mM ATP; Trace 3: CP + 1 mM ATP-γS; Trace 4: CP+ HSP18; Trace 5: CP + HSP18 preincubated with 1 mM ATP; Trace 6: CP + HSP18 preincubated with 1 mM ATP-γS. Each data point is the average of triplicate measurements. **Panels C and D** represents the percent protection ability of different HSP18 preparations against CS and lysozyme aggregation. The chaperone:client protein ratio was 1:1 (w/w) for CS and lysozyme aggregation assays. Data are the means ± standard deviation from triplicate measurements. **p< 0.005 and ***p<0.0005.

The effect of ATP on the chaperone function of *M*. *leprae* HSP18 is similar to that of other sHSPs whose chaperone function is increased by ATP [18–20]. But, whether ATP hydrolysis is required for enhancing the chaperone function of HSP18, needs to be explored. There are conflicting reports available in the literature regarding the requirement of ATP hydrolysis for modulation of chaperone function of different sHSPs. Muchowski and Clark reported that ATP hydrolysis is required for the improved chaperone function of α-crystallin and *M*. *tuberculosis* HSP16.3 [19–20], while Biswas and Das reported that the enhancement in the chaperone function of α-crystallin in presence of ATP was found to be independent of ATP hydrolysis [18]. Notwithstanding with this controversy, the chaperone function of *M*. *leprae* HSP18 was performed in the presence of non-hydrolysable analog of ATP i.e. ATP-γS. Fig. 5A and B (trace 5) show that at 1:1 (w/w) ratio of HSP18 to CS/lysozyme, in presence of 1 mM ATP, the chaperone function of HSP18 enhanced ~40% (Fig. 5C) and ~27% (Fig. 5D) respectively. When ATP was replaced with ATP-γS, the aggregation prevention ability of HSP18 was also enhanced and the extent of enhancement in chaperone function of this sHSP was almost similar to that of in the presence of ATP (Fig. 5A and B, trace 6). We also observed that the thermal deactivation prevention ability of HSP18 was enhanced in presence of 1 mM ATP-γS and MDH retained ~90% activity. This was identical when the solution contained 1 mM ATP (Fig. 4B). In light of all these observations, it is inferred that ATP hydrolysis is not required for the chaperone function of *M*. *leprae* HSP18.

### The interaction of ATP with HSP18 is reversible in nature

**Fig 6:**
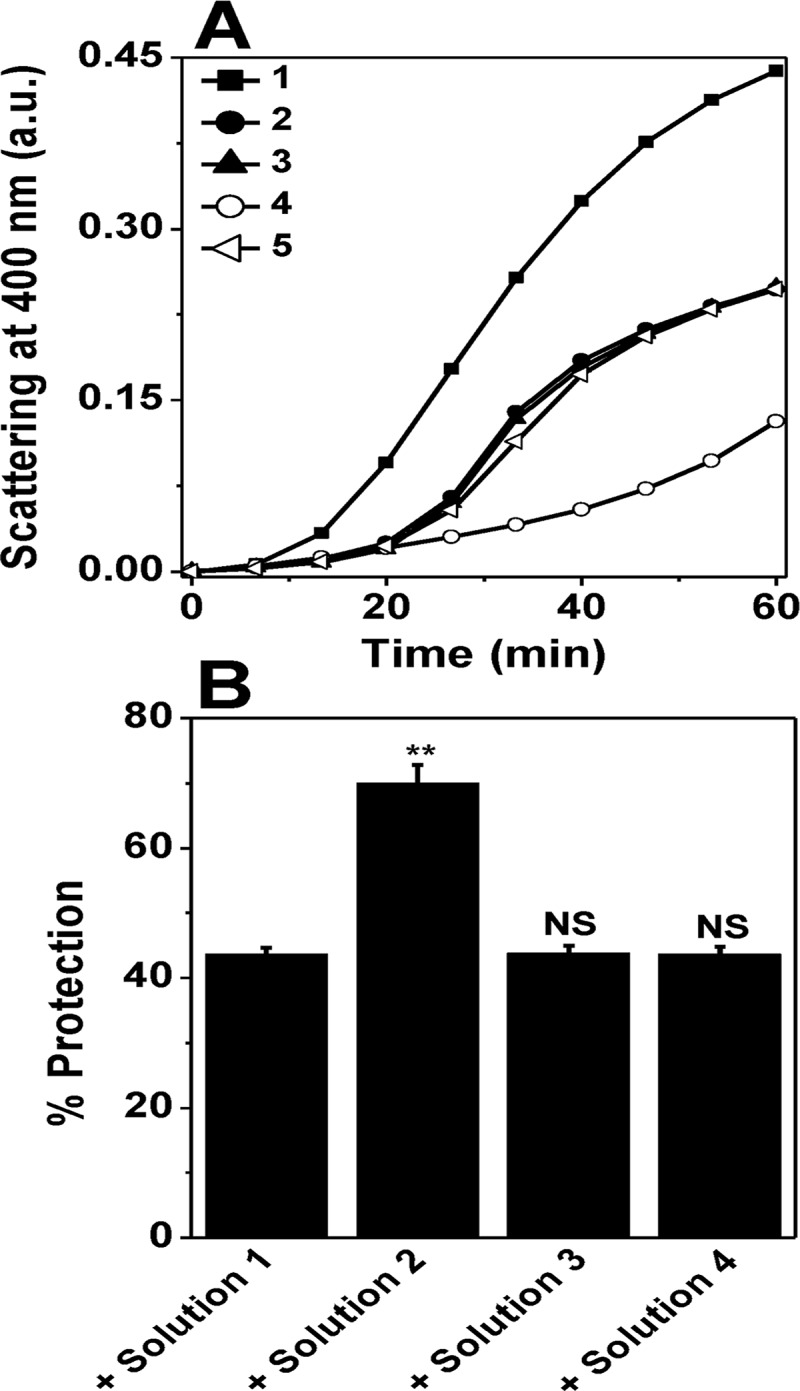
The alteration in the chaperone function of *M*. *leprae* HSP18 induced by ATP is reversible in nature. **(A)** DTT-induced aggregation of 0.05 mg/ml lysozyme (Lys) in the presence/absence of different HSP18 solutions. First, HSP18 was incubated in absence (solution 1) and presence of 1 mM ATP (solution 2) at 25°C for 1 hr. Then, part of solution 1 and 2 were dialysed overnight against 50 mM phosphate buffer, pH 7.5. The resultant solution achieved from the dialysis of solution 1 and 2 were termed as solution 3 and 4, respectively. Subsequently, assay was performed with these solutions. Trace 1: Lys alone; Trace 2: Lys + solution 1; Trace 3: Lys + solution 3; Trace 4: Lys + solution 2; Trace 5: Lys + solution 4. Lysozyme aggregation was initiated by adding DTT and scattering was measured while incubation of assay mixture at 37°C. **Panel B** represents the percent protection ability of different HSP18 solutions against lysozyme aggregation. The chaperone: client protein ratio used for this assay was 1:1 (w/w). Data are the means ± standard deviation from triplicate measurements. NS = Not significant and **p< 0.005.

The interaction between HSPs and ATP is often reversible in nature [18, 45]. To understand whether the modulation in the chaperone function of HSP18 in presence of ATP is reversible, we compared the aggregation prevention ability of different undialysed and dialysed HSP18 samples, details of which are mentioned in the figure legends. Fig. 6A (trace 2 and 4) and 6B reflects that HSP18 in absence and presence of 1 mM ATP prevented the aggregation of lysozyme by 23.6% and 62%, respectively at a 1:1 (w/w) chaperone to client protein ratio. Extensive dialysis had no effect on the chaperone function of HSP18. Overnight dialysed HSP18 showed almost identical chaperone activity to that of undialysed HSP18 (Fig. 6A, trace 3 and 6B). However, removal of ATP from the complex by overnight dialysis reduced the protection ability of HSP18 from 62% to 24% (Fig. 6A, trace 5 and 6B) which suggest that ATP induced alteration in the chaperone function of HSP18 is reversible in nature.

### ATP binding alters only the tertiary structure of HSP18

**Fig 7:**
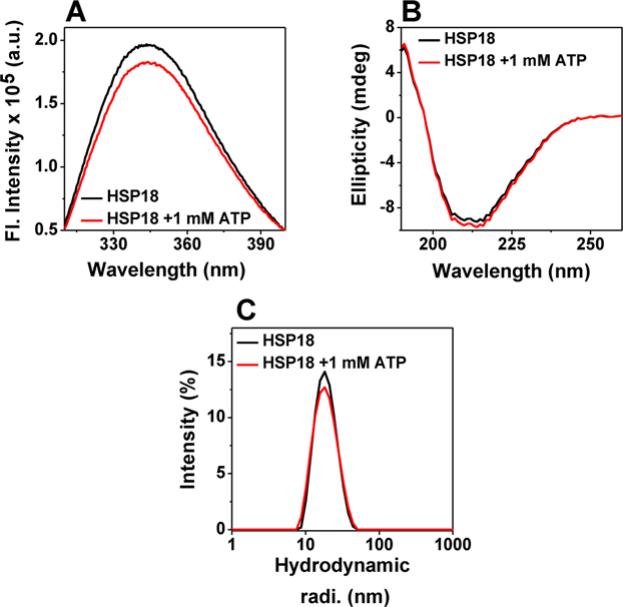
Effect of ATP on the structure/conformation of *M*. *leprae* HSP18. **(A)** Intrinsic tryptophan fluorescence spectra of HSP18 (0.05 mg/ml) in absence and presence of 1 mM ATP were recorded in the range 310–400 nm at 25°C. The excitation wavelength was 295 nm. Excitation and emission slit widths were 5 nm each. Data were recorded at a wavelength resolution of 0.5 nm. **(B)** Far-UV CD spectra of HSP18 in absence and presence of 1 mM ATP. Spectra were recorded for 0.2 mg/ml protein (in 10 mM phosphate buffer, pH 7.5) using a cell of 1 mm path length. The data interval was 1 nm. **(C)** Intensity particle size distribution spectra of *M*. *leprae* HSP18 were recorded at 25°C temperature in presence or absence of 1 mM ATP. Each of these spectra is an average of 48 scans.

Once it was confirmed that ATP altered the chaperone function of *M*. *leprae* HSP18, effect of ATP interaction on the conformation of this sHSP was examined. Tryptophan fluorescence, near- & far-UV CD and dynamic light scattering (DLS) techniques were used to determine whether the altered chaperone function was accompanied by any changes in tertiary, secondary and quaternary structure/conformation of the protein. The intrinsic tryptophan fluorescence of HSP18 decreased slightly (~7.5%) in presence of 1 mM ATP (Fig. 7A) which implied that tryptophan microenvironment was mildly perturbed due to the ATP interaction. Changes in tryptophan fluorescence have been also reported when ATP interacts with HSPs [16, 21]. The near UV-CD data also agreed with our tryptophan fluorescence data (S1C Fig.). Far-UV CD spectra of HSP18 in absence and presence of 1 mM ATP further suggested that ATP interaction did not perturb the secondary structure of this protein (Fig. 7B). We also used dynamic light scattering (DLS) technique to determine whether ATP interaction altered the quaternary structure i.e. oligomeric size of HSP18. HSP18 with or without 1 mM ATP exhibited a similar hydrodynamic radius of around ~18 nm (Fig. 7C) which indicated that ATP did not perturb the oligomeric/quaternary structure of *M*. *leprae* HSP18. Overall, these findings revealed that the secondary and quaternary structure of the protein remains unperturbed, while its tertiary structure undergoes mild alteration upon interaction with ATP.

### HSP18 becomes less susceptible towards tryptic cleavage in presence of ATP

**Fig 8:**
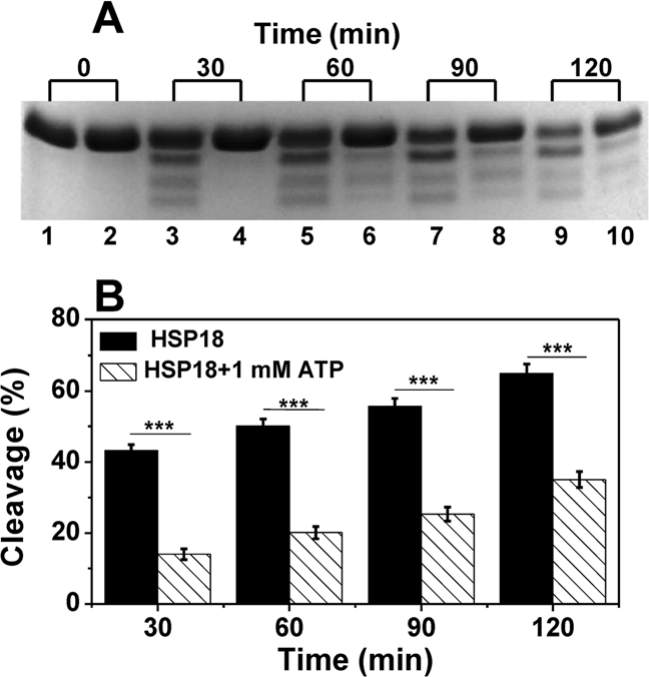
SDS-PAGE profile of trypsin digestion of *M*. *leprae* HSP18 in the absence and presence of 1 mM ATP. **(A)** HSP18 (0.5 mg/ml) in 50 mM phosphate buffer, pH 7.5 was digested with trypsin at 100:1 ratio (w/w) for different times at 37°C. All odd-numbered lanes had no ATP, and even-numbered lanes had 1 mM ATP. **(B)** Bar diagram representation of the net cleavage of *M*. *leprae* HSP18 by trypsin at different time points. ***p<0.0005

To achieve more knowledge about the conformational changes induced in HSP18 by ATP, we performed the trypsin digestion experiment. The SDS-PAGE profile of tryptic digestion products of HSP18 in absence or presence of 1 mM ATP at different digestion times using a 1:100 (w/w) ratio of trypsin to chaperone were shown in Fig. 8A. Trypsin considerably digested HSP18 after 120 min. in absence of ATP (lane 9), but the digestibility of HSP18 by trypsin retarded in presence of 1 mM ATP (lane 10). Moreover, densitometry analysis of trypsin mediated HSP18 cleavage experiment showed that, ~43% HSP18 cleavage occurred in absence of ATP even after 30 min digestion, while only 14% cleavage of HSP18 happened in presence of 1 mM ATP at the same time point (Fig. 8B). This effect of ATP, leading to decrease in tryptic cleavage of HSP18, is also seen with ATP-γS (S2 Fig.). These findings confirmed that ATP altered the protein conformation in a manner that trypsin cleavage sites became more shielded. This is in agreement with other findings where authors reported that ATP interaction made α-crystallin less susceptible towards tryptic cleavage [17–18].

### ATP induces additional exposure of hydrophobic patches at the surface of HSP18

**Fig 9:**
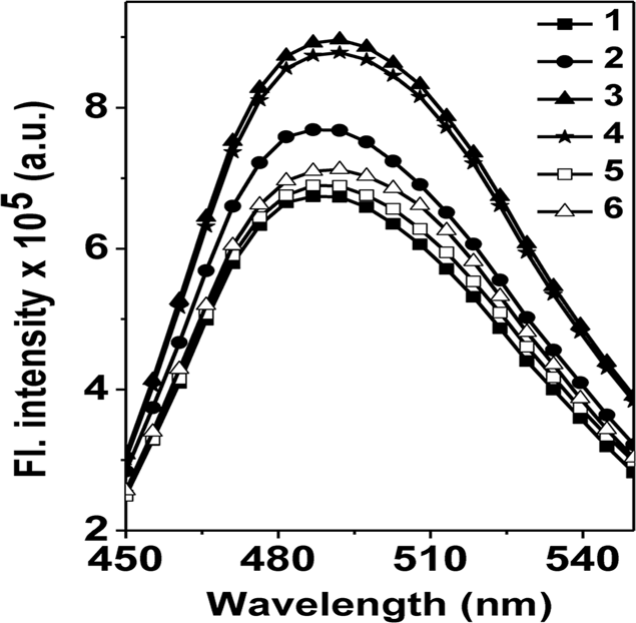
Effect of ATP on the surface hydrophobicity of *M*. *leprae* HSP18. First, HSP18 was incubated in absence (solution 1) and presence of presence of 0.01 mM ATP (solution 6)/1 mM ATP (solution 2)/1 mM ATP-γS (solution 5) at 25°C for 1 hr. Then, part of solution 1 and 2 were dialysed overnight against 50 mM phosphate buffer, pH 7.5. The resultant solution achieved from the dialysis of solution 1 and 2 were termed as solution 3 and 4, respectively. All these solutions were incubated with 10 μM bis-ANS at 25°C for 1 hr. The protein concentration was 0.05 mg/ml. The fluorescence spectrum of bis-ANS bound to different samples was recorded in the range 450–550 nm at 25°C. The excitation wavelength was 390 nm. Trace 1: bis-ANS + solution 1; Trace 2: bis-ANS + solution 6; Trace 3: bis-ANS + solution 2; Trace 4: bis-ANS + solution 5; Trace 5: bis-ANS + solution 3; Trace 6: bis-ANS + solution 4.

Tryptophan fluorescence and trypsin digestion experiments suggested that ATP exerted the conformational changes in HSP18. Apart from these conformational changes, the alteration in the surface hydrophobicity of HSP18 in presence of ATP was also determined. Both large and small HSPs bind aggregation prone substrate proteins through the available hydrophobic patches at the surface and it is generally believed that surface hydrophobicity governs the chaperone function of these HSPs [5, 18, 46–47]. Additionally, several reports in the literature showed that the enhancement in the chaperone function of sHSPs is often accompanied by increased surface hydrophobicity [5, 18, 47]. The surface hydrophobicity of HSP18 in absence and presence of 1 mM ATP/ATP-γS was estimated using bis-ANS. This hydrophobic probe has been widely used for probing the hydrophobic sites of several HSPs. We observed that the fluorescence intensity of bis-ANS bound to "ATP (0.01 mM) incubated HSP18" was ~ 16% higher compared to that of HSP18 alone (Fig. 9, trace 2). In presence of 1 mM ATP, the bound bis-ANS fluorescence intensity was increased further (~33%) (Fig. 9, trace 3). We also observed similar enhancement in the fluorescence intensity of bis-ANS bound to HSP18 in presence of 1 mM ATP-γS (Fig. 9, trace 4). Moreover, when we depleted the ATP from the solution by dialysis, the fluorescence intensity of bound bis-ANS reversed back and the magnitude was almost identical to the fluorescence intensity of bis-ANS bound to HSP18 alone (Fig. 9, traces 5–6). Altogether, our results confirmed that surface hydrophobicity of HSP18 was enhanced on interaction with ATP which resulted an increase in its chaperone activity. A similar explanation is also applicable for the enhanced chaperone activity of HSP18 in presence of ATP-γS. Furthermore, this conformational change is ATP hydrolysis independent and is reversible in nature. We also noticed that the change in the chaperone function of HSP18 in presence of ATP is also reversible in nature (Fig. 6B). This appears to be due to a reversible change in the surface hydrophobicity of HSP18.

### ATP binding enhances the structural stability of HSP18 against thermal and chemical denaturation

**Fig 10:**
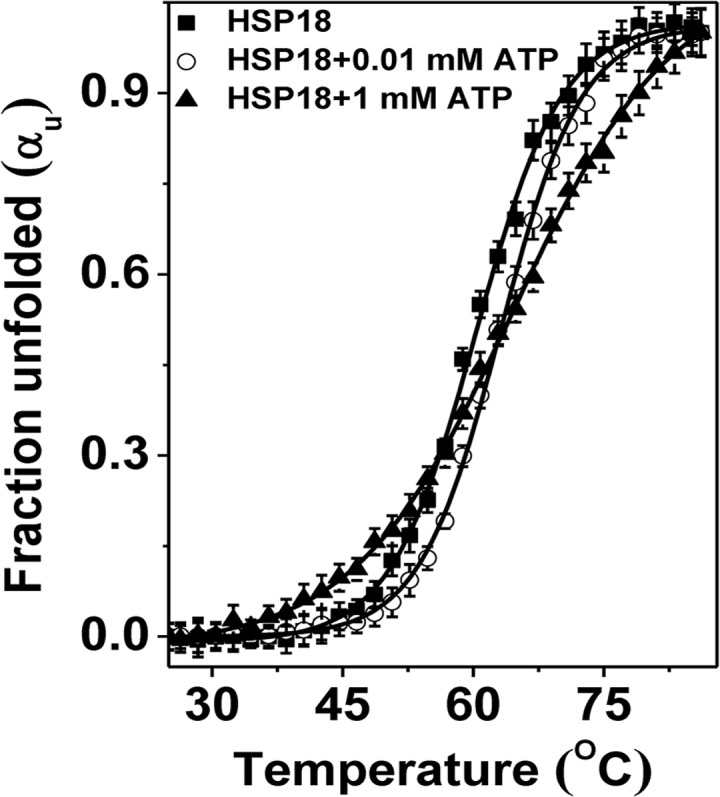
Effect of ATP on the structural stability of HSP18 against thermal stress. Temperature induced changes in the fraction of unfolded state (α_u_) for HSP18 in absence and presence of 0.01/1 mM ATP. The profile has been normalized to a scale of 0–1. Symbols represent the experimental data points and the solid lines represent the best fit according to the sigmoidal analysis.

Since ATP triggered exposure of additional hydrophobic sites on the surface of *M*. *leprae* HSP18, it was a genuine curiosity that, whether this additional exposure of hydrophobic sites was caused by the formation of a molten globule-like intermediate structure or by the reorganization of hydrophobic patches at the surface of HSP18. A molten globule is typically a partially unfolded intermediate state of a protein, with a native-like secondary structure but grossly perturbed/disordered tertiary structure and a significant exposure of hydrophobic surface [48]. Although, HSP18 possess native-like secondary structure in presence of ATP, the mild perturbation in the tertiary structure upon ATP interaction denies the chances of formation of molten globule-like structure of HSP18 in presence of ATP. Also, the formation of this partially unfolded molten globule-like structure would have decreased the structural stability of HSP18. But, the resistance towards the trypsin digestion in presence of ATP (Fig. 8) indicated that ATP possibly made the structure of HSP18 more compact or stable. To understand the alteration in structural stability induced by ATP properly, we compared the structural stability of *M*. *leprae* HSP18 in absence or presence of 0.01/1 mM ATP against thermal stress using far-UV CD measurements. The change in the ellipticity magnitude at 222 nm were monitored over a temperature range from 25 to 85°C and then fraction unfolded (α_U_) for HSP18 in absence or presence of ATP were calculated using the following equation:

$$a_{U}=\frac{\theta_{F}-\theta_{T}}{\theta_{F}-\theta_{U}}$$ (3)

where, θ_F_ is the ellipticity value at 25°C for completely folded or native protein, θ_T_ is the observed ellipticity value at any temperature and θ_U_ is the ellipticity value at 85°C for the completely denatured or unfolded state. We plotted fraction unfolded (α_U_) as a function of temperature (Fig. 10). The thermal denaturation profiles of HSP18 in absence and presence of 0.01/1 mM ATP were sigmoidal in nature and exhibited an apparent two state transition. Sigmoidal analysis of these profiles (Fig. 10, solid lines) demonstrated that HSP18 alone (in absence of ATP) underwent thermal unfolding with a midpoint transition or melting temperature (T_m_) of ~60.3°C. In presence of 0.01 and 1 mM ATP, the T_m_ value shifted to 63.1°C and 64.5°C, respectively (Fig. 10). The increase in the mid-point transition or melting temperature (T_m_) value of HSP18 under thermal stress by ~3–4°C clearly suggests that ATP increased the thermal stability of *M*. *leprae* HSP18.

**Fig 11:**
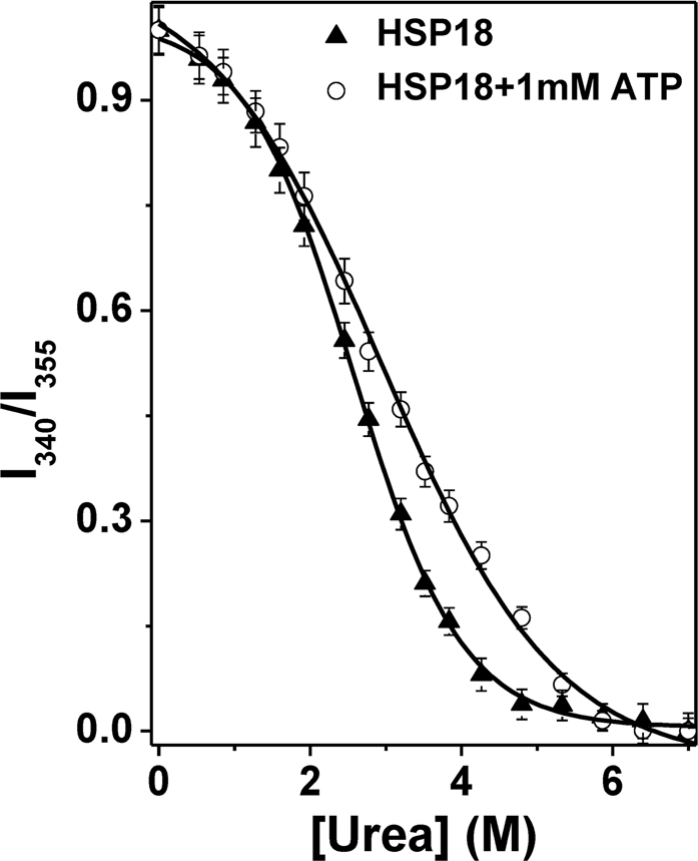
Effect of ATP on the thermodynamic stability of *M*. *leprae* HSP18. Equilibrium urea unfolding profile for 0.05 mg/ml of HSP18 in the absence and presence of 1 mM ATP at 25°C. The profile has been normalized to a scale of 0–1. Symbols represent the experimental data points and the solid lines represent the best fit according to the three state model described by Equation 4.

We also examined the thermodynamic stability of HSP18 in absence and presence of 1 mM ATP. Equilibrium urea unfolding was estimated by monitoring the intrinsic tryptophan fluorescence of both the system at various urea concentrations. The λ_max_ values were recorded at 340 nm and 355 nm and plotted as a ratio of intensities (I_340_/I_355_) against urea concentration (Fig. 11). Crude estimation of the transition midpoint (*C*_1/2_) from sigmoidal analysis of the denaturation profiles indicated that the *C*_1/2_ value increased from 2.57 M for HSP18 alone to 2.90 M of urea for HSP18 in presence 1 mM ATP (Fig. 11 and Table 1). This increase suggested that ATP stabilized the overall structural integrity of HSP18. To quantify the stability, all of the profiles were analyzed with the aid of a global three-state fitting procedure, according to the following equation:

$$F=\frac{F_{N}+F_{I}\exp\left(-\Delta G_{1}^{0}+m_{1}\left[urea\right]\right)/RT+F_{U}\exp\left(-\Delta G_{2}^{0}+m_{2}.\left[urea\right]\right)/RT}{1+\exp\left(-\Delta G_{1}^{0}+m_{1}\left[urea\right]\right)/RT+\exp\left(-\Delta G_{2}^{0}+m_{2}.\left[urea\right]\right)/RT}$$ (4)

where F_N_, F_I_ and F_U_ are the fluorescence intensities for 100% native, 100% intermediate and 100% unfolded forms, respectively. ΔG_1_° represents the standard free energy change between native (N) and the intermediate (I) forms, and ΔG_2_° represents the standard free energy change between the intermediate (I) and unfolded (U) forms. ΔG°, which is the sum of ΔG_1_° and ΔG_2_°, represents the standard free energy change of unfolding (between the N and U forms) at zero urea concentration. The standard free energy change of HSP18 in absence of ATP at 25°C was 20.77 kJ/mol (Table 1). In presence of 1 mM ATP, the ΔG° value for HSP18 was increased to 24.78 kJ/mol, suggesting an increase in the thermodynamic stability by ~4 kJ/mol. Though this difference is small but reproducible. Both chemical and thermal denaturation experiments further excluded the possibility of formation of molten globule-like structure of HSP18 in presence of ATP. Rather, ATP interaction gave rise to an energetic ladder of higher stability which is not far from the native state and has originated due to the native state minor fluctuations. Some researchers also observed the formation of such energetic ladder in various proteins. Specific mutations in T4 lysozyme produced energetic ladder [49]. Similarly, interaction of α-crystallin with ATP also formed energetic ladder [18]. In both cases, creation of energetic ladder with slightly higher stability improved the chaperone function of α-crystallin. Therefore, we can conclude that higher structural stability of HSP18 upon ATP interaction is a basis for the enhancement of its chaperone function.

**Table 1:** C_1/2_ and ΔG values of *M*. *leprae* HSP18 in absence and presence of 1 mM ATP at 25°C.

System Studied	C_1/2_ (M)	ΔG° (kJ/mol)
HSP18	2.57 ± 0.12	20.77 ± 0.45
HSP18+1mM ATP	2.9 ± 0.13	24.78 ± 0.53

### Determination of Walker-B ATP binding motif and probable ATP binding sites in *M*. *leprae* HSP18

**Fig 12:**
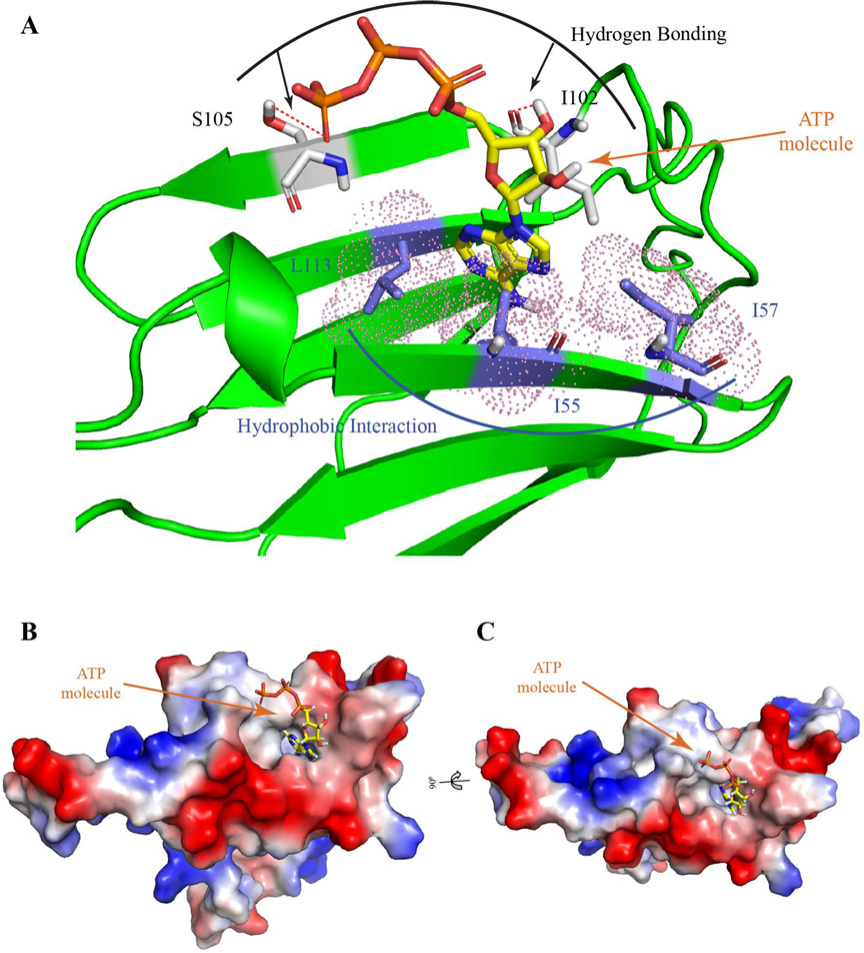
Representation of the modeled three-dimensional structure of HSP18 when docked with ATP molecule. **(A)** Ribbon representation of docked complex i.e. HSP18 with ATP molecule. Key residues involved in forming hydrogen bonding with ATP molecule are shown in white color code and those involved in making hydrophobic contacts are shown in blue color code. **(B and C)** Representation of vacuum generated electrostatic potential of protein surface in presence of ATP. The electropositive surface/region is represented with blue color, electronegative surface/region is represented with red color and hydrophobic surface/region is presented with white color (with mesh representing the van der Waal radii). The terminal phosphate group (γ-PO_4_) of ATP molecule is found to be in close proximity of electropositive counters.

Finally, we took an attempt to get an idea about the “ATP binding motif” in *M*. *leprae* HSP18. Previously, Ghosh *et al*. reported the “ATP binding motif” in human αB-crystallin [50]. Comparative sequence alignment of αB-crystallin with 19 ATP binding proteins, identified the sequence, _82_KHFSPEELKVKVLGD_96_ as the Walker-B ATP binding motif which lies in the “α-crystallin domain” of αB-crystallin. Interestingly, *M*. *leprae* HSP18 also belongs to small heat shock protein family and possess an “α-crystallin domain” (S3 Fig.). Sequence homology between *M*. *leprae* HSP18 and αB-crystallin showed that they share ~51% sequence similarity. Besides, “α-crystallin domain” of both the proteins was found to share ~57% sequence similarity (S3 Fig.). Therefore, primary sequence of *M*. *leprae* HSP18 was aligned and compared with that of αB-crystallin, which contained the Walker-B ATP binding motif, R/K/H-X_2-10_-O-X-O-D/E, where X is any amino acid and O is any hydrophobic amino acid (A/C/G/F/I/L/M/V/W). Based on sequence similarity, the sequence _49_KADSLDIDIE_58_ of *M*. *leprae* HSP18 in its “α-crystallin domain” has been identified as the Walker-B ATP binding motif. The three dimensional structure of HSP18 (obtained through the homology modeling using coordinates of HSP16.9, PDB code: 1GME_A) indicated that part of this motif lies in β4 strand and is followed by a loop (Fig. 12), which is in agreement with the structure of Walker-B ATP binding motif found in different ATP binding proteins.

Since, we observe a high sequence similarity between αB-crystallin and HSP18, we hypothesized that the binding site for ATP in HSP18 would be similar to that of αB-crystallin. ATP usually binds to β4-β8 groove in αB-crystallin [50]. To test this hypothesis, first, the three dimensional structure of HSP18 was obtained through the homology modeling using coordinates of HSP16.9 (PDB code: 1GME_A) and was validated with the help of Ramachandran plot (S4 Fig.). S4 Fig. shows the distribution of phi (ϕ) and psi (ψ) angles of the protein, where majority of amino acid residues are found to be in allowed region. After successful validation of the structure of HSP18, docking of ATP was performed which revealed that ATP molecule binds to the similar groove/strands in HSP18 as was found in αB-crystallin. The terminal γ-phosphate group of ATP molecule interacts with the side chain of Ser105 which lies in β8 strand of HSP18 (Fig. 12). On the other hand, the adenine moiety of the molecule was found to be in close proximity of Ile55 and Ile57 of Walker-B motif which reside in another β strand i.e. β4 strand (Fig. 12). Moreover, side chains of few residues from β8 strand were found to be in close proximity to the α- and β-phosphate groups of ATP molecule. This figure also suggests that there is a possibility of hydrogen bonding between the side chains of Ser105 with γ-phosphate group of ATP, while the interaction between the adenine moiety of ATP molecule and Ile55/Ile57/Leu113 is hydrophobic in nature. The docking studies also showed that hydrogen bonding may also be possible between the carbonyl group of main chain of Ile102 residue and the hydroxyl group of sugar moiety of ATP (Fig. 12). During docking studies, we also estimated the total energy (potential and kinetic energy) of HSP18 in absence and presence of ATP. The total energy for the protein was found to be −2.8 × 10^4^ kJ/mol, whereas the total energy for docked complex was found to be −3.2 × 10^4^ kJ/mol. These results suggested that the structure of HSP18 is stabilized upon the interaction with ATP, which is in agreement with the findings of our thermal and chemical denaturation experiments (Figs. 10 and 11). Recently, Biswas and his coworkers showed that acetylation of γD crystallin destabilized its structural stability and was accompanied with lower potential energy compared to that of wild type γD-crystallin [51]. Additionally, we also took an attempt to understand how the local environments near to the ATP binding sites (_49_KADSLDIDIE_58_ which contains residues of β4 strand and _102_ILASYQE_108_ which contains residues of β8 strand) are modulated upon ATP binding. We estimated the solvent accessible surface area (SASA) of each residue of these two stretches (Table 2). Before addition of ATP, the total SASA for hydrophobic and hydrophilic residues in _49_KADSLDIDIE_58_ were 196.58 and 541.07 Å^2^ for full residue, respectively, while the same for side chain were 105.09 and 505.88 Å^2^, respectively. In docked complex, the total SASA for hydrophobic residues in _49_KADSLDIDIE_58_ was increased (230 and 141.65 Å^2^ for full residue and side chain, respectively), while the same was decreased for hydrophilic residues (517.59 and 464.65 Å^2^ for full residue and side chain, respectively). We observed the similar trend in the other stretch (_102_ILASYQE_108_) as well. Therefore, we can say that the hydrphobic nature of local environments increases upon ATP binding. In bis-ANS experiment, we also found that the availability of hydrophobic patches at the surface of oligomeric HSP18 enhances in presence of ATP (Fig. 9). Overall, these theoretical studies demonstrated that ATP binds to β4-β8 groove/strands in HSP18, but some parts of this small molecule are at the surface of the groove and thus exposed towards the solvent.

**Table 2:** Solvent accessible surface area (SASA) of hydrophobic and hydrophilic residues in the local environments of HSP18 subunit near to the ATP binding sites (_49_KADSLDIDIE_58_ which contains residues of β4 strand and _102_ILASYQE_108_ which contains residues of β8 strand) in absence and presence of ATP.

	Residues	Total ASA without ATP (Å^2^)		Total ASA with ATP (Å^2^)	
		Full residue	Side chain	Full residue	Side chain
Hydrophobic residues present in _49_KADSLDIDIE_58_	A50, L53, I55 and I57	196.58	105.09	230	141.65
Hydrophillic residues present in _49_KADSLDIDIE_58_	K49, D51, S52, D54, D56 and E58,	541.07	505.88	517.59	464.65
Hydrophobic residues present in _102_ILASYQE_108_	I102, L103 and A104	220.87	159.3	232.65	201.13
Hydrophillic residues present in _102_ILASYQE_108_	S105, Y106, Q107 and E108	470.12	409.67	469.52	408.13

In summary, the present study demonstrates that *M*. *leprae* HSP18 interacts efficiently with ATP only, and not with ADP and AMP. ATP possibly binds to the β4-β8 groove/strands in HSP18 and the binding affinity of HSP18 towards ATP is moderate. This moderate interaction can be concluded from the fact that the binding between HSP18 and ATP is reversible in nature. As a result of such moderate reversible interaction, minor alterations in the conformational states and stabilization energy of the protein are observed which eventually gives rise to a thermodynamically distinct state, so called energy ladders. We believe that it is the ATP induced microscopic fluctuations and shifting of energy states of HSP18 trigger the exposure of additional hydrophobic sites at the surface of protein and thus improves its chaperone function. Several reports have revealed that the molecular chaperone function of HSP18 may play a vital role in the growth and survival of the pathogen in host macrophages [6, 22]. Out of these reports, one study demonstrated that the expression level of HSP18 increases under different stressed conditions such as hypoxia, nutrient depletion, oxidative stress, thermal stress etc. [22]. In the same paper, it has been shown that HSP18 could provide tolerance to cells under such stressed conditions. As reversible binding of ATP to *M*. *leprae* HSP18 enhances its chaperone function without any significant alteration in its conformations, such association process can be of significant importance in order to protect itself as well as the pathogen under several physiological stressed environments.

Recently, ATP-competitive inhibitors are widely used as drugs for the treatment of another tropical disease i.e. tuberculosis [52–55]. Wolfe *et al*. have used a probe based proteomics approach to find out ATP-binding proteome of *M*. *tuberculosis*, a causative organism of tuberculosis and identified 122 ATP-binding proteins [56]. Most of these proteins are critical for the continued existence of *M*. *tuberculosis* and may be appropriate therapeutic targets for development of ATP-competitive antibiotics. Among these 122 proteins, *M*. *tuberculosis* HSP16.3, a class 1 small heat shock protein, also showed binding affinity towards ATP. Interestingly, the chaperone function of this protein is also enhanced in presence of ATP [19] and it is generally believed that the chaperone function of HSP16.3 is important for the survival of *M*. *tuberculosis* pathogen in the latent stationary phase of tuberculosis [19]. Since, *M*. *leprae* HSP18 possess ~55% sequence homology with HSP16.3 and its chaperone function also enhances in presence of ATP, *M*. *leprae* HSP18 may possibly be an important therapeutic target in leprosy. Use of ATP competitive antibiotics/inhibitors especially against HSP18 could be of significant importance in context of the effective treatment of leprosy, but these aspects needs to explored extensively.

## Supporting Information

S1 Fig ATP interacts with *M*. *leprae* HSP18 and the effect of this interaction on its structure and function. **(A)** SPR analysis of the interaction of ATP (0.1–5 μM) to immobilized *M*. *leprae* HSP18 interactions. The ATP solution was prepared in degassed, filter-sterilized PBS buffer without 3.5 mM MgCl_2_. The binding affinity of this interaction was found with a dissociation constant (K_d_) value of 0.56 μM. **(B)** Percent protection ability of *M*. *leprae* HSP18 against CS aggregation in the absence and presence of 0.2/0.5 mM ATP at 43°C. Data are means ± the standard deviation from triplicate determinations. **p< 0.005 and ***p< 0.0005. **(C)** Near-UV CD spectra of HSP18 in absence and presence of 1 mM ATP. Spectra were recorded for 0.5 mg/ml protein (in 50 mM phosphate buffer, pH 7.5) using a cell of 10 mm path length. The data interval was 0.5 nm.
(TIF)

S2 Fig SDS-PAGE profile of trypsin digestion of *M*. *leprae* HSP18 in the absence and presence of 1 mM ATP-γS. **(A)** HSP18 (0.5 mg/ml) in 50 mM phosphate buffer, pH 7.5 was digested with trypsin at 100:1 ratio (w/w) for different times at 37°C. All odd-numbered lanes had 1 mM ATP-γS, and even-numbered lanes had no ATP-γS.
(TIF)

S3 Fig Sequence alignment of *M*. *leprae* HSP18 and αB-crystallin. The amino acid sequence alignment between αB-crystallin and HSP18 was performed using multiple sequence alignment software ClustalW. *, identical residues;:, conserved substitutions;., semiconserved substitutions. The "α-crystallin domain" of both proteins share ~57% sequence similarity.
(TIF)

S4 Fig Validation of the modeled three dimensional structure of HSP18 using Ramachandran plot. Ramachandran plot of HSP18, with psi (Ψ) and phi (ϕ) angle representing the Y- and X-axis scale, where majority of the amino acid residues are found within the allowed region.
(TIF)
