# Supplementary figures and images for: Interaction of ATP with a Small Heat Shock Protein from Mycobacterium leprae: Effect on Its Structure and Function
Source: PLoS Negl Trop Dis. 2015 Mar 26;9(3):e0003661. doi: 10.1371/journal.pntd.0003661 (PMC4374918; doi:10.1371/journal.pntd.0003661)

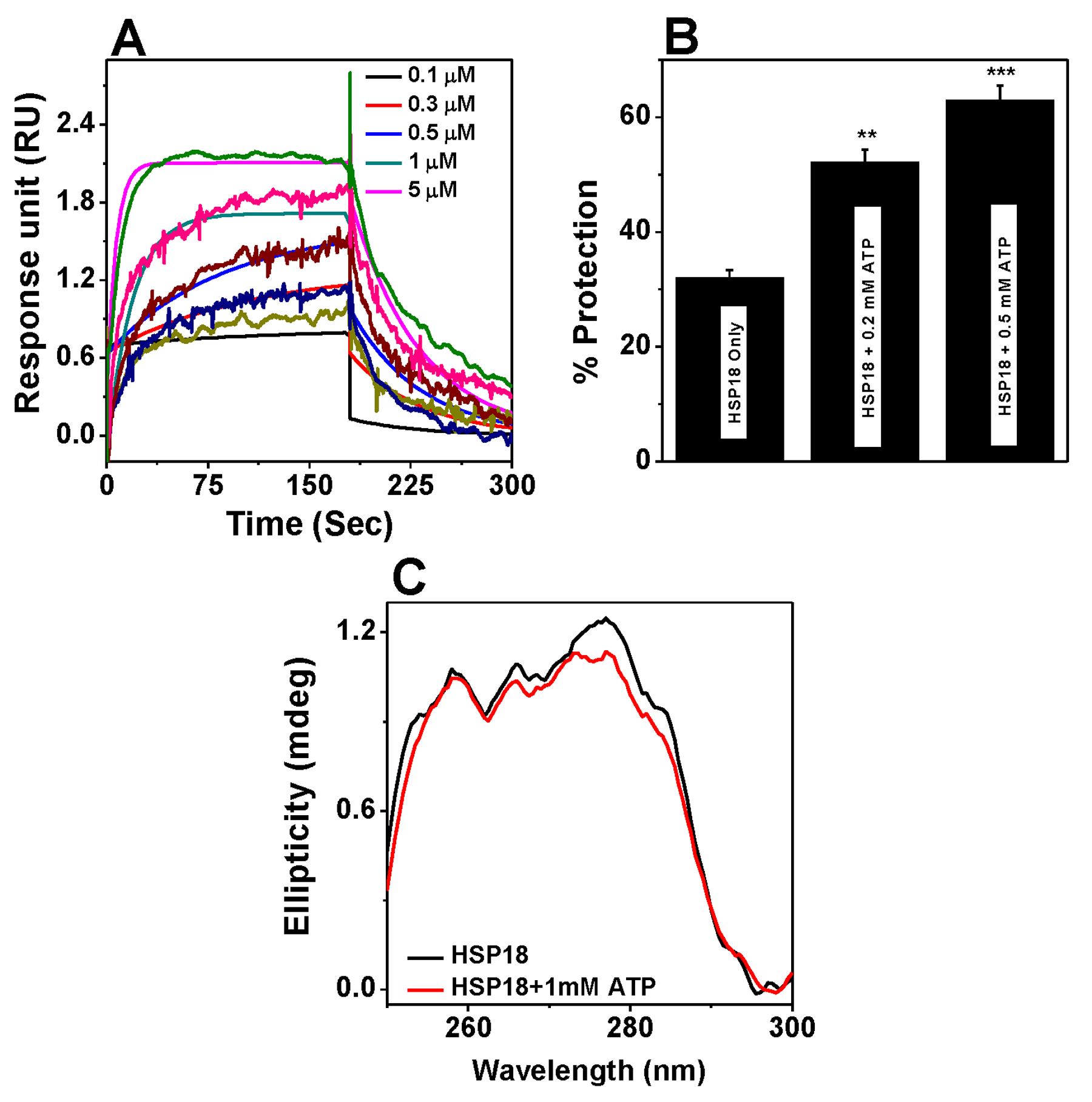

Supplement: S1 Fig — (A) SPR analysis of the interaction of ATP (0.1–5 μM) to immobilized M. leprae HSP18 interactions. The ATP solution was prepared in degassed, filter-sterilized PBS buffer without 3.5 mM MgCl2. The binding affinity of this interaction was found with a dissociation constant (Kd) value of 0.56 μM. (B) Percent protection ability of M. leprae HSP18 against CS aggregation in the absence and presence of 0.2/0.5 mM ATP at 43°C. Data are means ± the standard deviation from triplicate determinations. **p< 0.005 and ***p< 0.0005. (C) Near-UV CD spectra of HSP18 in absence and presence of 1 mM ATP. Spectra were recorded for 0.5 mg/ml protein (in 50 mM phosphate buffer, pH 7.5) using a cell of 10 mm path length. The data interval was 0.5 nm. (TIF) [file pntd.0003661.s001.tif]

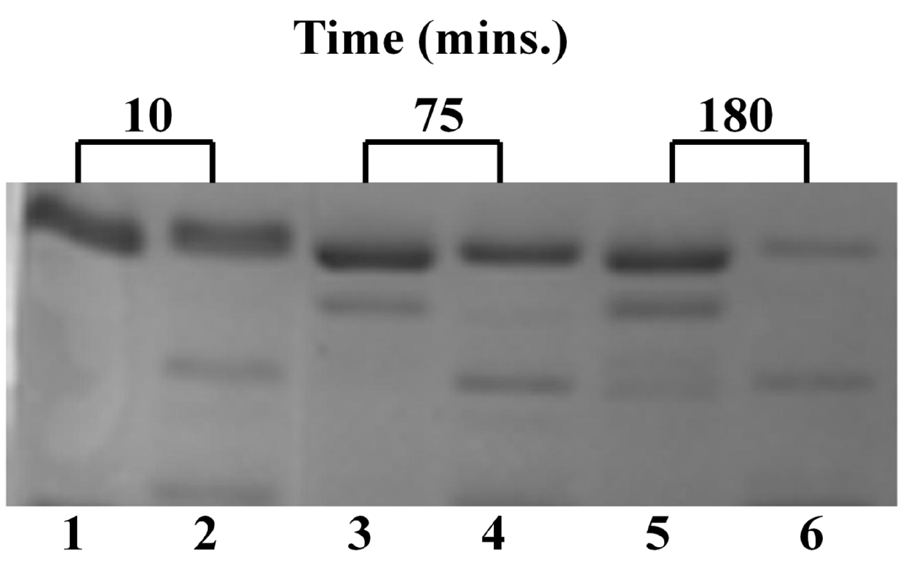

Supplement: S2 Fig — (A) HSP18 (0.5 mg/ml) in 50 mM phosphate buffer, pH 7.5 was digested with trypsin at 100:1 ratio (w/w) for different times at 37°C. All odd-numbered lanes had 1 mM ATP-γS, and even-numbered lanes had no ATP-γS. (TIF) [file pntd.0003661.s002.tif]

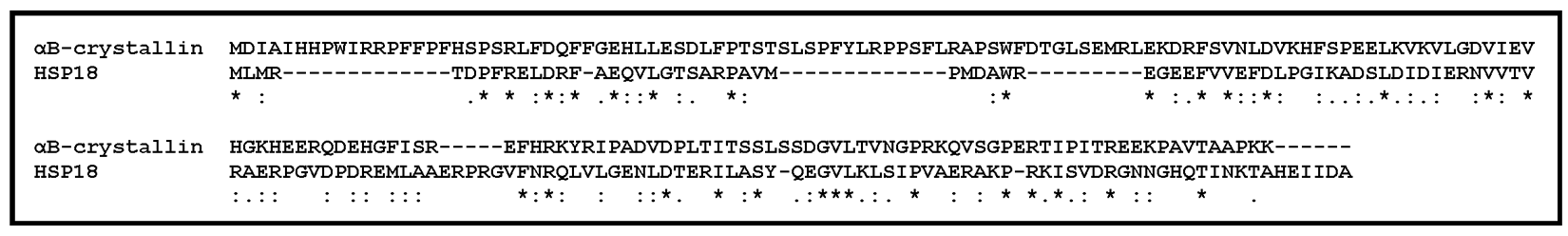

Supplement: S3 Fig — The amino acid sequence alignment between αB-crystallin and HSP18 was performed using multiple sequence alignment software ClustalW. *, identical residues;:, conserved substitutions;., semiconserved substitutions. The "α-crystallin domain" of both proteins share ~57% sequence similarity. (TIF) [file pntd.0003661.s003.tif]

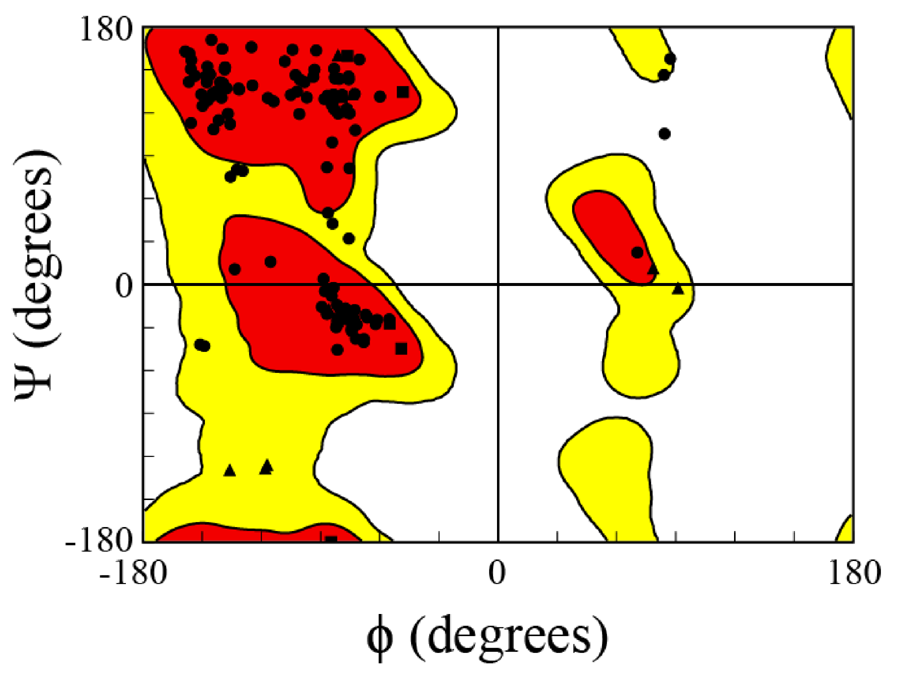

Supplement: S4 Fig — Ramachandran plot of HSP18, with psi (Ψ) and phi (ϕ) angle representing the Y- and X-axis scale, where majority of the amino acid residues are found within the allowed region. (TIF) [file pntd.0003661.s004.tif]
